# Assessment of human resources for health programme implementation in 15 Latin American and Caribbean countries

**DOI:** 10.1186/s12960-015-0016-4

**Published:** 2015-04-28

**Authors:** Mario Roberto Dal Poz, Hernan Rodrigo Sepulveda, Maria Helena Costa Couto, Charles Godue, Monica Padilla, Rick Cameron, Thais de Andrade Vidaurre Franco

**Affiliations:** Institute of Social Medicine, University of the State of Rio de Janeiro, Rua São Francisco Xavier, 524, Pavilhão João Lyra Filho, 7 andar / bloco D, Maracanã, Rio de Janeiro CEP 20550-013 Brazil; Human Resources for Health Programme, Pan American Health Organization, Washington, USA; Human Resources for Health, Pan American Health Organization, San Salvador, El Salvador; Cameron Health Strategies Group, Halifax, Nova Scotia Canada

**Keywords:** Americas, Health evaluation, Health manpower, Health plan Implementation, Health programmes and plans

## Abstract

**Background:**

The health systems in the Americas region are characterized by fragmentation and segmentation, which constitute an important barrier to expanding coverage, achieving integrated primary health care, and reducing inefficiency and discontinuity of care.

An assessment of the human resources for health (HRH) programmes that have been implemented at the country level was developed as part of the measurement of the 20 HRH regional goals for 2007–2015, adopted in 2007 by the Pan American Sanitary Conference (CSPA).

**Methods:**

The exercise was a combination of academic research and the development/application of an advocacy tool involving policy makers and stakeholders to influence the decision-making in the development, implementation, or change of HRH programmes while building evidence through a structured approach based on qualitative and quantitative information and the exchange and dissemination of best practices.

**Results:**

This paper covers the methodological challenges, as well as a summary of the main findings of the study, which included 15 countries: Belize, Costa Rica, El Salvador, Guatemala, Honduras, Nicaragua and Panama in the Central America, Dominican Republic in the Caribbean, Chile, Colombia, Ecuador and Peru in the Andean sub region, and Argentina, Paraguay, and Uruguay in the South Cone. Despite the different contexts, the results showed that the programmes evaluated faced common challenges, such as lack of political support and financial unsustainability.

**Conclusions:**

The evaluation process allowed the exchange and dissemination of practices, interventions, and programmes currently running in the region. A shared lesson was the importance of careful planning of the implementation of programmes and interventions. The similarities in the problems and challenges of HRH among the participating countries highlighted the need for a cooperation programme on the evaluation and assessment of implementation strategies in the Americas region.

## Abstract in Spanish

Los sistemas de salud en la región de las Américas se caracterizan por la fragmentación y la segmentación, lo que constituyen una importante barrera a la expansión de la cobertura, el logro de la atención primaria de salud, y la reducción de la ineficiencia y la discontinuidad de la atención.

Una evaluación de los programas de recursos humanos para la salud (RHS) implementados a nivel nacional fue desarrollada como parte de la medición de las 20 metas regionales para los RHS en el periodo 2007-2015, aprobadas por la Conferencia Panamericana Sanitaria (CSPA) en 2007.

El ejercicio fue una combinación de investigación académica y desarrollo/aplicación de un instrumento de promoción involucrando los responsables políticos y las partes interesadas para influir en la toma de decisiones acerca de desarrollo, implementación o modificación de los programas de RHS, mientras favorecer el aumento del conocimiento a través de un enfoque estructurado basado en información cualitativa y cuantitativa y en el intercambio y la difusión de las mejores prácticas.

Este documento cubre los desafíos metodológicos, así como un resumen de las principales conclusiones del estudio, que incluyó a 15 países: Belice, Costa Rica, El Salvador, Guatemala, Honduras, Nicaragua y Panamá en América Central, República Dominicas en el Caribe, Chile, Colombia, Ecuador y Perú en la subregión andina y Argentina, Paraguay y Uruguay en el Cono Sur. A pesar de los diferentes contextos, los resultados mostraron que los programas evaluados enfrentan desafíos comunes, como la falta de apoyo político y de insostenibilidad financiera.

El proceso de evaluación ha permitido el intercambio y la difusión de las prácticas, las intervenciones y los programas actualmente en ejecución en la región. Una lección compartida fue la importancia de la planificación cuidadosa de la aplicación de los programas e intervenciones. Las similitudes en los problemas y desafíos de los RHS entre los países participantes destacaron la necesidad de un programa de cooperación en la evaluación y valoración de las estrategias de implementación en la región de las Américas.

## Background

In the last decade, the central role of the workforce in improving the health sector has become evident. Several studies have identified signals in the relationship between the quantity and quality of workers and the expansion and qualification of access to health services. Despite this, however, there is still a crisis in the health workforce, characterized by a shortage of professionals, an inadequate skill mix, and an unequal distribution of professionals [1,2].

In response to this crisis, there is a growing demand for countries to develop and implement programmes for the management and planning of human resources for health (HRH). The interventions aim to confront complex issues, such as the migration of health professionals, the need to make the health system more responsive to demographic changes and the ageing population, and the challenge of ensuring the presence of professionals in remote and rural locations [1].

Moreover, the failure of health system reforms has been associated with the failure to strengthen the policies, planning, and management of HRH early in the process. In a systematic review, significant gaps were found in knowledge about the way training, regulatory, financial, and organizational mechanisms affect the supply, distribution, and performance of health care workers. Additionally, the data available tend to come from high-income settings and may not apply to low- and middle-income countries (LMICs) [3].

According to Peters et al. [4], despite the need for information to support decisions on policies, programmes, and practices in health, the implementation research is a growing but not well-understood field. Implementation research seeks to understand what, why, and how interventions work in “real environments” and to determine the possible approaches to improve them.

This article describes the process and results of the evaluation of health human resources programmes developed through cooperation between the Pan American Health Organization (PAHO) and the Institute of Social Medicine, State University of Rio de Janeiro. The study covered 15 countries in Latin America Belize, Costa Rica, El Salvador, Guatemala, Honduras, Nicaragua and Panama in the Central America, Dominican Republic in the Caribbean, Chile, Colombia, Ecuador and Peru in the Andean sub region, and Argentina, Paraguay, and Uruguay in the South Cone.

The programmes’ evaluation started from concerns raised in the monitoring process of the 20 regional goals for human resources for health for 2007–2015 adopted by the Pan American Sanitary Conference in 2007 [5]. The purpose of the evaluation was to develop collaborative strategies in the Americas; gather information about the development of HRH programmes to support decision-making in the formulation, implementation, or modification of health policies; and expand and maintain a workforce able to support primary health care, with the aim of expanding the coverage of national health systems.

The HRH programmes’ evaluation at the regional level could also allow sharing of information and trends, identifying emerging issues, and promoting opportunities for future collaboration. Because of this cooperation process, different partners can exchange information about best practices. The evaluation was developed not only as an academic research but also to contribute to changes and the improvement of HRH programmes and policies, by empowering and encouraging the creation of a network.

The evidence and reflections presented in The World Health Report 2006 [1] and the compromise established around the development of the “decade of human resources” mobilized countries, agencies, associations, and international organizations and influenced a number of actions for international collaboration on the subject of HRH. In the Americas, the favourable international scenario for the recognition of HRH issues was added to the already existing network of observatories of human resources established in 1999. In 2005, the PAHO promoted the Seventh Regional Meeting of the Observatories of Human Resources in Health, which originated the “Toronto Call to Action for a Decade of Human Resources in Health for the Americas (2006-2015)” [6]. This initiative took place in parallel with the development of a strategy of decentralization of technical cooperation in four sub regions: the Andean, the Southern Cone, Central America, and the Caribbean, which was conducted by PAHO in agreement with the governments of the region to recognize the dynamics of integration and the similarities between countries [7].

At the 27th Pan American Health Conference, 20 regional goals to be achieved by 2015 were established in the process of transforming health systems towards the renewal of primary health care and the integration of services. The goals were grouped according to the five critical areas mentioned in the Toronto Call to Action: 1) define policies and long-term plans, 2) improve the distribution of professionals, 3) regulate professional flows and migration, 4) create healthy work environments, and 5) establish links between training institutions and services [6].

The Toronto Call to Action in 2005 and, thereafter, the Regional Goals for Human Resources for Health 2007–2015 have established a strategic framework to direct efforts in HRH management, recognizing the importance of HRH planning and guidance of health systems based on primary health care and universal coverage. To monitor the 20 regional goals, a methodology was developed that allowed establishing a baseline and identifying the needs and progress in every area covered in the goals, as well as recognizing the priorities to be addressed in the implementation of HRH public policies. The evaluation process of the HRH programmes analysed in this article is part of this context of international cooperation, information exchange, and dissemination of practices and interventions.

## Methods

The approach proposed had three main components, completed in accordance with the possibilities in the different countries included in the evaluation. The first step was to identify and list the programmes and interventions that each country has implemented or is implementing to address HRH recruitment, deployment, and retention concerns and problems. As a result, it has built an inventory of programmes to be analysed and studied in depth, resulting in evidence on the challenges in HRH management and policy.

The second step started with a description of the programme, including the identification of indicators of programme results from government reports and other data sources. A programme description conveys the mission and objectives of the programme being evaluated. It should be sufficiently detailed to ensure understanding of the goals and strategies of the programme, its capacity to effect change, its stage of development, and how it fits into the larger organization and community. The description enables comparisons with similar programmes and facilitates attempts to connect programme components to their effects [8,9].

The aspects included in the programme description were need, expected effects, activities, resources, stage of development, context, and a logic model [10-13]. A logic model described the sequence of events for bringing about change by synthesizing the main programme elements into a picture of how the programme is supposed to work. One of the virtues of a logic model is its ability to summarize the overall mechanism of change by linking processes to eventual effects. A logic model can also display the infrastructure needed to support programme operations [14]. Further, it reveals assumptions concerning the conditions for programme effectiveness and provides a frame of reference for one or more evaluations of the programme [15,16].

To obtain more details on the programme-implementation process and its results, a formal questionnaire was used to collect data from secondary sources but also to interview key informants. Additionally, the information gathered was used to complete a logic model for the purpose of identifying and linking all major components and mapping the process of programme implementation.

In the third step of this study, a template was developed for the country report to put together all the information gathered and to analyse the processes and results of the programme in relation to the expected outputs and outcomes.

**Figure 1:**
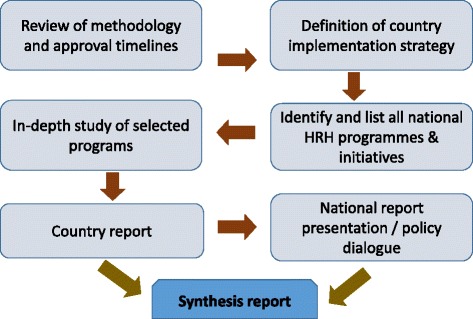
Steps in assessing the HRH programmes.

The stages of the programme evaluation are interdependent; that is, the success of each step is dependent on the success of the step before it. Therefore, there was an effort to clearly define and agree on programme goals, activities, and indicators, trying to assure the stakeholder participation and acceptance. Figure 1 summarizes the steps in assessing the HRH programmes and strategies implemented at the national level.

The respective country authorities, along with the national offices of PAHO/WHO, selected the national assessment teams. To support these teams in the preparation, data collection, interviews, and so on, an agreement was made with the PAHO/WHO Collaborating Centre on Health Workforce Planning and Information at the Institute of Social Medicine at the University of the State of Rio de Janeiro (IMS/UERJ). This made possible the conduct of face-to-face and virtual meetings with national teams to support the programme evaluation process. Although the basic process and objectives were the same in all countries, it was possible to take into account the capabilities of each country and allow flexibility in the way they deepened their evaluation [4]. For the purpose of this project, the following operational definition was used:

*A****programme****is defined as a long*-*term* (*more than 1 year*) *formal plan of action*, *with a sequence of actions that describe how a health system or service will operate*, *including details such as roles and responsibilities*, *planned expenditures*, *results*, *etc. Less formal*, *and generally shorter*-*term than a programme* (*one year and less*), *an****initiative****is defined as a coherent set of actions*, *activities or interventions that are linked to an ultimate goal and one or more policy or programme objectives and have certain resources* (*human*, *financial*, *material*) *to meet the objectives and contribute to the achievement of the goal*.

After listing the programmes and initiatives, the following criteria were used to select the programmes to be evaluated: at least one HRH programme/initiative on HRH, effect (impact) on the health system, total amount of programme funding, scope of the programme (population and geographic area), temporary extension (over a year), and the availability of information.

Results related to changing partner and stakeholder attitudes and behaviour as a factor for the successful development, implementation, and management of programmes were only marginally addressed.

## Results

Although the studied countries differ significantly in their political, social, economic, and geographic contexts, the HRH programmes and initiatives showed that the actions of these countries have been directed to strengthening national health systems, oriented towards primary health care with universal and equitable coverage. Beyond that, many of the challenges faced in the implementation of human resource programmes are the same across countries; these include the difficulty of training HRH, the lack of political will and financial support, weak HRH information systems, fragile/weak HRH governance, and difficulties in hiring and retaining health professionals in the public sector and in their allocation in remote and rural areas.

Issues related to the programme design were described as challenges by some countries. For instance, in Honduras, most of the programme development is not based on any previous design, and there is no regular discussion regarding the adaption of the programme to human resources training and the health needs of the population. Another example is Chile, where the biggest challenge for programme design is the appropriate planning of the project. In El Salvador, there is still little consistency between HRH programmes and health needs.

The fragility of intermediate and long-term planning remains a challenge in achieving the overall HRH objectives in countries such as Costa Rica, Nicaragua, Ecuador, and Argentina. In El Salvador, Costa Rica, Guatemala, Honduras, Panama, Dominican Republic, and Argentina, programme implementation was described as a big challenge, especially when extending the programme to the national level.

The lack of clarity of roles was identified by Paraguay and Honduras as a challenge in programme development. The need for different authorities to understand their new roles in the programme was mentioned in Honduras. In Paraguay, there is a need to clarify and improve the coordination among different actions, programmes, and institutions to avoid overlap or inattention. The overlapping of actions by different participants or institutions also exists in Panama. Challenges at the participant level were reported in El Salvador, Costa Rica, Guatemala, Honduras, Dominican Republic, Chile, and Panama.

Low acceptance of the programme by professionals was reported in Guatemala, where medical specialty residents partially rejected the programme, and in the Dominican Republic, where doctors struggled to be evaluated on their performance. The resistance of professionals to changes in the health system was highlighted in El Salvador, whereas in Honduras, low adherence to the changes by the community was found, in addition to resistance among professionals.

In Guatemala, the Health Secretary was considered as a key player, whereas in Nicaragua, the importance of an integrated approach to planning HRH policies was stressed. In Argentina and Paraguay in Mercosur and in Chile, Ecuador, and Peru in the Andean region, the importance of the planning process was also highlighted. Peru cited the value of planning in expanding the access and coverage of health care.

The countries identified the importance of community participation and an intersectoral approach in implementing HRH programmes. Guatemala, Chile, and Peru emphasized the importance of a common vision among stakeholders and cooperation among technical and academic institutions towards the establishment of a shared responsibility among stakeholders. In El Salvador, intersectoral work (interagency cooperation), albeit slow, was reported to be effective.

The set of programmes presented are aligned with the five challenges of the Toronto Call to Action. Tables 1 and 2 present the key contents of the evaluated programmes and the issues addressed in the sub regions by each country.

**Table 1:** **Key contents of the selected programmes by country and region**

**Region**	**Countries**	**Programme areas**
America Central	Panamá	PHC strengthening, HRH training, training of specialists, and continuing education
	Honduras	Family health (integration between social work students and universities and community health teams) and implementation of PHC services
	Dominican Republic	Training of PHC teams, training of public health directors, and recruitment to strengthen PHC
	Guatemala	Training of specialists, university midwifery training, curriculum review and training, and establishment of health careers
	El Salvador	Human resource planning as a tool for implementing PHC and training of managers and technical teams
	Belize	PHC provided by community health workers recruited from the community
	Nicaragua	Organization of a decentralized HRH management system, implementation of a new health delivery model with community participation, and PHC training of support staff (nursing assistants)
	Costa Rica	Strategic nurse training, PHC strengthening, and training of specialists
Mercosur	Argentina	HRH information, permanent health education, joint intersectoral health education, and adapting the training of specialists to regional needs
	Paraguay	Family health unit expansion guided by the PHC and training of family physicians (virtual training)
	Uruguay	Increasing the number and distribution of nurses, increasing the number of skilled nurses/physicians, changes in work organization, and changes in labour relations
Andean	Chile	Training of specialists according to the needs of the population
	Peru	Community service done by qualified and registered health professionals, training of medical specialists, and training for PHC development with emphasis on family and community health at the primary care level
	Ecuador	Return of health professionals to the country to fulfil the requirements of critical areas, job security planning, and training of medical specialists and subspecialists with affirmative actions allowing incorporating persons belonging to minorities
	Colombia	Improving the distribution of specialists in the country

**Table 2:** **Themes and programmes by country and region**

**Theme**	**Programmes**	**Central America**								**Andean region**				**Mercosur**		
		**BEL**	**ELS**	**CRI**	**GUA**	**HON**	**NIC**	**PAN**	**DOR**	**ECU**	**COL**	**CHI**	**PER**	**ARG**	**PAR**	**URU**
Training	Training/specialization/permanent education	X	X	X	X		X	X	X	X	X	X	X	X	X	X
	Increase of health professionals, technicians, and specialists		X	X	X		X	X		X	X	X	X	X	X	
	Training of public health managers								X							
	Establishment and/or strengthening of PHC	X	X	X	X	X	X	X	X				X		X	
	Curriculum revision				X											
Labour management	Career path/contractual agreements			X	X					X						X
	HRH planning		X													
	Management decentralization						X									X
	HRH information													X		
	Migration									X						
	Distribution of health professionals, technicians, and specialists		X		X		X	X		X	X	X	X	X		
	Other themes													X		

In seven countries, the analysed programme is related to the first challenge of the Toronto Call to Action: “To define policies and long-term plans on human resources”. From this viewpoint, El Salvador has established a planning process for organizing primary health care and a programme for training managers. These programmes were made possible by strengthening the institutional capacity with the establishment of the Department of Human Resources Development.

All the countries have addressed the second challenge of the Toronto Call to Action to “place the right people in the right places” through initiatives and programmes related to professional training. Almost all the countries have made efforts to increase the number of specialists in different medical fields and to strengthen primary health care (PHC), indicating the concern of local governments with the training of health professionals and technicians.

The *Cierre de Brechas* plan in Chile provided funding for the training of medical specialists who were committed to provide public health services in areas with critical shortages. The *Sistema Nacional de Acreditación de Residencias del Equipo de Salud* programme in Argentina involves the establishment of minimum requirements for the medical residency system, with places and priorities defined according to regional needs. Panama, Guatemala, Costa Rica, Peru, Ecuador, and Colombia also presented programmes aimed at training specialists to meet the needs of the population.

The strengthening of PHC or its implementation through HRH training was related to programmes in the eight countries in the Central American region. In Guatemala, the *Formación de Técnicas Universitários de Partería* programme meets the challenge of reducing maternal and neonatal deaths by training people to integrate health teams in their own communities.

The third challenge of the Toronto Call to Action focused on the regulation of health worker flows and migration. This evaluation found seven countries trying to address the HRH self-sufficiency issue in HRH policy.

Ecuador was the only country that developed a programme directly related to the migration of health professionals. The said programme, *Ecuador Saludable*, *Vuelvo Por Ti*, began in 2012 and aimed to promote the return of health professionals who were abroad to cover gaps in critical specialties and areas. A website was initiated for registering professionals working abroad who were interested in returning to the country; these professionals were offered professional stability and specialization programmes. By the end of 2013, about 700 professionals had been hired through the programme, and the experience brought by these professionals was highlighted for its contribution in enriching the sector.

Five countries addressed the fourth challenge of the Toronto Call to Action, which addressed the need to “promote healthy work environments and commitment to the institutional mission of ensuring quality health services for the entire population”. Ecuador targets 80% of health workers by 2017 with its labour stability plan, whereas Uruguay started in 2012 a programme to reform medical services, with the aim of improving the quality of care, prioritizing primary care, and enhancing the quality of life of medical professionals.

Seven countries assessed programmes related to the fifth challenge, which involves developing interaction mechanisms between training institutions and health services to improve the training of health workers (Target 17). For instance, in 2012, the Dominican Republic developed a programme in agreement with the Universidad Autónoma de Santo Domingo, aiming to train human resources based on the primary health care model.

Because the “Toronto challenges” are correlated, the evaluation naturally found that the programmes are also connected to more than one challenge, thus reinforcing the correlation.

Communication mechanisms and changes in organizational culture were identified as a development strategy in Argentina and El Salvador, particularly when it comes to managing the HRH. Regarding budget support, Honduras, Ecuador, Argentina, and Chile reported that support in terms of resources facilitates the implementation of programmes. The importance of information as support was identified in Ecuador, Argentina, and El Salvador.

Costa Rica reported that the implementation of the programme taught them the importance of the nursing profession. Honduras identified as interesting the lesson that the empowerment of health workers is a key factor in the implementation of a new health delivery model because of their management capacity.

The Central American countries highlighted the importance of training in strengthening primary health care. In El Salvador, Costa Rica, and Honduras, the need to promote closer ties between health training and practice was emphasized. In Guatemala, the need to strengthen supervision to improve the quality of training programmes was stressed. Training health professionals was also a challenge in Argentina, Chile, Panama, and Ecuador.

The lack of health professionals with adequate skills and competences, especially in PHC, was described as a challenge in Belize, Honduras, and Paraguay. Aiming to increase health coverage and ensure the quality of training and services, 14 of the 15 countries had programmes and initiatives to promote integration between educational institutions and health services, with the specific goal of improving the skills, capabilities, and training of doctors, nurses, and other technical professionals focused on primary health care and different specialties.

Argentina, Uruguay, and Ecuador also cited the lessons learned regarding staff training, but these were not assessed in this study.

In Central America, Dominican Republic identified IT development as a good practice that allows the systematization of administrative and financial procedures concerning staff contracts. Costa Rica recognized the importance of professional regulation and a competitive recruitment process. Peru, Paraguay, and El Salvador reported good practices related to changes in health care, including comprehensive care, the importance of teamwork, and community and family health models. The importance of teamwork was also reported in El Salvador. Meanwhile, Paraguay cited employment status and compensation as ways to reduce moonlighting.

Lack of financial sustainability and challenges regarding the availability of information for the planning, implementation, or continuity of the programme were also identified. Difficulties related to the programme budget was highlighted and described in El Salvador, Belize, Costa Rica, Guatemala, Honduras, Panama, Ecuador, Chile, Argentina, and Uruguay. Such financial constraint was responsible for restricting the maintenance and expansion of programmes and for obstructing the establishment and implementation of a national HRH policy.

Just as decisions related to HRH cannot be considered only by using economic criteria, so without adequate funding, it is not possible to address the health needs of the population, achieve the agreed-on goals, and guarantee the remuneration of health workers. For instance, in Costa Rica, there is an understanding that there are insufficient financial resources to address the health needs of the population and strengthen HRH. Honduras does not have a defined budget for human resources development. Ecuador had to postpone the first payments to those health workers who returned to the country from abroad, causing huge dissatisfaction. Further, Uruguay limited the number of new areas in the training programme due to lack of financial resources. Because of the weakness of the processes and the difficulty of developing projects or of integrating programmes within health systems, programmes and projects often develop in isolation, dependent on the availability of donor funds and without connection to the system as a whole.

In many countries, such as Argentina, Guatemala, and El Salvador, the need to establish work agreements with educational institutions was mentioned. In addition, the identification of key stakeholders and the creation of institutional spaces of negotiation (e.g. in Guatemala) can compensate for the lack of cooperation between these institutions and the lack of involvement of key partners in HRH programmes.

The study results showed that the lack of supporting data and information remains a critical challenge for El Salvador, Costa Rica, Guatemala, Honduras, Dominican Republic, Chile, Argentina, and Paraguay. For instance, the importance of improving the data quality and of training staff on data collection tools was mentioned in Paraguay, whereas, in Argentina, local managers had difficulty updating the HRH data regularly.

An increase in the use of virtual platforms in distance training programmes was observed. For instance, in Argentina, this sort of technology has been used to train professionals and tutors in a virtual campus for public health.

In Costa Rica and Chile, retaining health professionals in the public sector was reported as a challenge, whereas El Salvador mentioned the burden of migration and high turnover of HRH. The difficulty of retaining health professionals in rural areas was also a challenge in Panama, El Salvador, and Paraguay. In Belize, this challenge is exacerbated because of the geography, low-population density, and scattered population, which make it even more difficult to organize health care services in remote and inaccessible areas. Difficulties in ensuring access to health services, increasing coverage, reducing waiting times, providing health care 24 h a day, and decreasing PHC gaps were considered as challenges in Nicaragua, El Salvador, Dominican Republic, and Ecuador.

## Discussion

The concerns found in this research are similar to the ones noted by Ranson [2] and Dussault et al. [17] when discussing the HRH policy concerns and research priorities in low- and middle-income countries.

Understanding the nature of policy implementation and looking at this process can shed light on the barriers to and facilitators of more effective implementation [18]. Therefore, any evaluation process needs to consider the difficulties that have been experienced and the lessons that have been learned in the implementation of HRH programmes and interventions.

Implementation is an ongoing process of decision-making by key actors, who work in complex policy and institutional contexts and face pressures from interested as well as opposing parties. As such, the motivation, flow of information, and balance of power and resources among stakeholders influence the policy-implementation processes [18].

In this study, most of the challenges involved in several phases and elements along the programme-to-action continuum were related to planning and programme design, stakeholder engagement, context, resources, and operational issues, which shape the decisions and actions at various levels.

The initial stages of HRH programme development, such as the design and implementation planning, are crucial processes. According to Bhuyan et al. [18], the programme content should frame clearly the underlying problem area, the policy goals and objectives, and the beneficiary population. Unclear or confusing objectives or actions may be one reason why some policies are not implemented.

Another key issue in programme implementation is the involvement of and coordination with the stakeholders. These stakeholders include the groups or individuals responsible for the implementation, the people affected by the programme, and the officials and professionals accountable for achieving the programme goals. The involvement of stakeholders in the implementation can be challenging because this often requires “joint actions” in response to new partnerships that did not exist previously [18,19].

The insufficient commitment of key participants to implement programmes also emerged as a challenge. In some cases, stakeholder groups and organizations are not always committed to the same outcomes and must reach an agreement to support the implementation. As the programme implementation unfolds, additional stakeholders may find themselves being affected by the changes and may seek to also insert themselves into the process [18].

To be feasible, HRH planning and implementation require the involvement of stakeholders, along with a solid financial basis and the necessary resources, such as time, skills, technology, equipment, information, and others. The programme descriptions highlighted situations in which a mismatch existed between the planned activities and the resources available for their implementation.

Intersectionality was identified as a key issue for HRH planning to provide comprehensive and quality health services. Most of the programmes analysed seemed quite comprehensive, in terms of including training institutions, ministries of health, professional associations, and community associations as key stakeholders in the process of changing and reforming health systems and delivery models.

Other difficulties in implementing HRH programmes include the following: lack of motivation for teamwork, unqualified recruitment mechanisms, outdated laws, unregulated health services provision, absence of impact measurement on the quality of care programmes, unmonitored activities, low educational level of candidates from rural communities, and nursing workload (rotating and moonlighting schedules).

Although some of the initiatives and programmes evaluated were related to health training and labour management, almost all the countries considered success to be related more to the achievement of changes in the health delivery model and the reduction of HRH and health inequalities. Despite the current challenges, the countries have made progress in increasing the coverage of primary health care especially in the most vulnerable areas, providing vocational training, and strengthening the planning and establishment of a national human resources policy.

## Conclusions

This study provides information on a set of HRH programmes currently being developed in the region, including the challenges and lessons learned by each country at the national and regional levels. The value of evaluation as an institutional practice is a factor that was noted by all the countries and that needs to be promoted. The study results’ are consistent with the findings from other studies and authors [20-22].

It is interesting to note that, although much is being done towards the achievement of health care goals, the gap between what can be done and what is being done is increasing. The success in reducing this gap will be determined largely by the successful development of the health workforce in alignment with the improvement of health systems, including on health system governance and financing.

The relevance and importance of the evaluation process and the need for its institutionalization should also be emphasized, having shown its benefits and proven its relevance, as well as the need to enhance international cooperation in this area [23].
